# Macrophage accumulation within coronary arterial wall in diabetic patients with acute coronary syndrome: a study with in-vivo intravascular imaging modalities

**DOI:** 10.1186/s12933-020-01110-8

**Published:** 2020-09-05

**Authors:** Takaaki Kogo, Takafumi Hiro, Daisuke Kitano, Tadateru Takayama, Daisuke Fukamachi, Tomoyuki Morikawa, Mitsumasa Sudo, Yasuo Okumura

**Affiliations:** Division of Cardiology, Department of Medicine, Nihon University School of Medicine, 30-1 Oyaguchi-Kamicho Itabashi-ku, Tokyo, 173-8610 Japan

**Keywords:** Acute coronary syndrome, Diabetes mellitus, Macrophage, Optical coherence tomography, Shear stress

## Abstract

**Background and aims:**

Macrophage accumulation in arteriosclerotic plaque of coronary arteries is involved in plaque destabilization. Atherosclerosis has been known to be progressive in patients with type 2 diabetes mellitus (DM). This study compared the features of 3-dimensional (3D) spatial distribution of macrophage accumulation within coronary artery wall between acute coronary syndrome (ACS) patients with DM (n = 20) and those without (non-DM, n = 20) by using intravascular ultrasound (IVUS) and optical coherence tomography (OCT).

**Methods:**

The OCT-derived macrophage accumulation was measured within the proximal left anterior-descending artery. This measurement was performed for the whole vessel segment of interest, higher shear stress region (flow divider side) and lower shear stress region (the opposite side).

**Results:**

Normalized macrophage accumulation per unit length of the whole segment of interest was significantly larger in ACS patients with DM than without. In non-DM patients, macrophage density per IVUS-derived plaque volume was significantly higher in high shear stress region compared to low shear stress region, however, there was no significant difference between the two regions in DM patients. The macrophage density in the low shear stress region was significantly higher in the DM group than in the non-DM group. A multivariate analysis showed that the presence of DM was a major determinant for macrophage distribution.

**Conclusions:**

Macrophage accumulation was more abundant and homogeneous within coronary arterial wall in DM patients with ACS compared to non-DM patients, suggesting that plaque destabilization may occur more widely throughout coronary wall in DM patients.

## Background

The main cause of acute coronary syndrome (ACS) is rupture of coronary plaque followed by thrombus formation [1]. Macrophage has been reported to play an important role during various steps of atherosclerosis up to the plaque rupture [2–7]. Macrophages within plaque derived from migrated monocytes or smooth muscle cells phagocytose oxidized low-density lipoprotein (LDL) to become foamy cells and to form fatty streak and lipid core [8]. Macrophage secretes a wide variety of chemicals including IL-1β provoking local inflammation or matrix metalloproteinase which causes thinning of fibrous cap leading to higher vulnerability of plaque rupture [3–5]. Pathological examination of patients who died by ACS showed the involvement of inflammation accompanied by macrophages in both plaque rupture and erosion [6]. In addition, plaques of ACS patients have been shown to have a higher density of macrophages than plaques of stable angina pectoris [7]. In particular, those atherosclerotic processes have been more progressive in diabetes mellitus (DM) patients [9].

Macrophage infiltration into the vessel wall usually has localized heterogeneity throughout vessel wall partly due to heterogeneity of shear stress distribution. Several clinical and in vivo small animal studies of histological or multi-modality assessment of large and medium arteries have reported that macrophage infiltration can be observed more frequently in regions where shear stress is relatively higher, including an upstream side across stenotic plaque [10–14]. Nonetheless, whether or not underlying disease such as diabetes mellitus (DM) affects the distribution of macrophage infiltration within coronary arterial wall has not been clarified. Recently, intravascular optical coherence tomography (OCT) is considered to enable detect macrophage accumulation within coronary arterial wall [15–18]. Some previous studies have revealed with OCT that DM patients more likely have macrophage infiltration within coronary wall [19, 20], however, stereoscopic features of macrophage accumulation along the wall were not fully clarified. Therefore, the major purpose of the present study was to examine specific features of macrophage infiltration within coronary arterial wall in patients with ACS having type-2 diabetes mellitus (DM) using in vivo intravascular imaging modalities.

## Methods

### Study patients

This study was a single-center, retrospective, non-randomized study conducted from 2011 to 2017, which selected patients who met the following criteria: (1) Patients diagnosed as the first ACS to undergo coronary angiography (CAG) and percutaneous coronary intervention (PCI) at Nihon University Itabashi Hospital, (2) patients who underwent intravascular ultrasound (IVUS) and optical coherence tomography (OCT), and (3) patients who had a distinct OCT-derived coronary macrophage accumulation in the range of 20 mm from the entrance of the left anterior descending branch (LAD) (immediately after the left circumflex branch). ACS was defined as ST-elevation type acute myocardial infarction, non-ST elevation type myocardial infarction and unstable angina pectoris where there was no responsible lesion throughout the LAD. Patients with a history of coronary artery disease more than one month ago, those with a history of obvious stroke, chronic heart failure requiring treatment, aortic disease, or kidney disease, dialysis patients, those with liver disease, malignancy, thyroid diseases, familial hypercholesterolemia or poorly controlled hypertension, as well as those who had regularly use steroids or nonsteroidal antiinflammatory drugs during blood purification, immunization/chemotherapy including LDL apheresis were all excluded. Since the medical insurance system in Japan allowed us to use IVUS and OCT for the same patient only when IVUS imaging alone was so poor in image quality to perform percutaneous coronary interventions of the culprit lesion, the selection of patient examined was considerably limited. As a result, this study consisted of 40 patients, 20 patients with DM and 20 patients without it. The presence or absence of diabetes was judged from HbA1c value at hospitalization of 6.5% or more, from the use of diabetes medication, and presence of the past history that diabetes was diagnosed by an attending physician. No patients with impaired glucose tolerance, which was a diabetes precursor condition, were included. All patients examined provided written informed consent for this study. This study was performed in accordance with the 1975 Declaration of Helsinki and approved by the Institutional Review Board of the Itabashi Hospital of Nihon University School of Medicine.

### IVUS and OCT procedure

After performing regular CAG in the cardiac catheter examination, a guide wire was inserted to the periphery of the left anterior descending artery, and the following intravascular imaging was performed via the guide wire before PCI. Immediately before this test, 1.5 mg of isosorbide dinitrate was injected into coronary artery. The observation range was from the periphery as far as possible through the entrance portion of LAD. Withdrawing of the imaging modalities was performed with an automatic constant speed. As for IVUS, OptiCross ™ Imaging Catheter (iLab ™ System, manufactured by Boston Scientific, Marlborough, MA) was used. The withdrawing speed was 0.5 mm/sec, and its frame-rate was 30 frames/sec. For OCT, Dragonfly ™ JP Imaging Catheter (ILUMIEN ™, OPTIS ™, St. Jude Medical, St. Paul, MN, USA) was used. The OCT imaging speed was 40 mm/sec and the imaging frame-rate is 180 frames/sec. Cases in which the quality of the image is bad, those with calcification at an elevation angle of 90 degrees or more within the measurement range are inappropriate for analysis were excluded. The vascular segment to be analyzed was selected as long as possible within 20 mm in length from the entrance of LAD. The following measurements of IVUS and OCT were performed blinded to clinical data.

### IVUS measurement

In the Gray scale IVUS, the cross-sectional areas of vessel and lumen were first measured for every 0.5 mm interval within the segments of interest, and the plaque area was then calculated from the difference between the two areas [21]. The summation of the cross-section areas multiplied by 1 mm in length was calculated to obtain the total vessel volume, the total lumen volume, and the total plaque volume.

### OCT measurement and three-dimensional analysis of macrophage accumulation

**Fig. 1:**
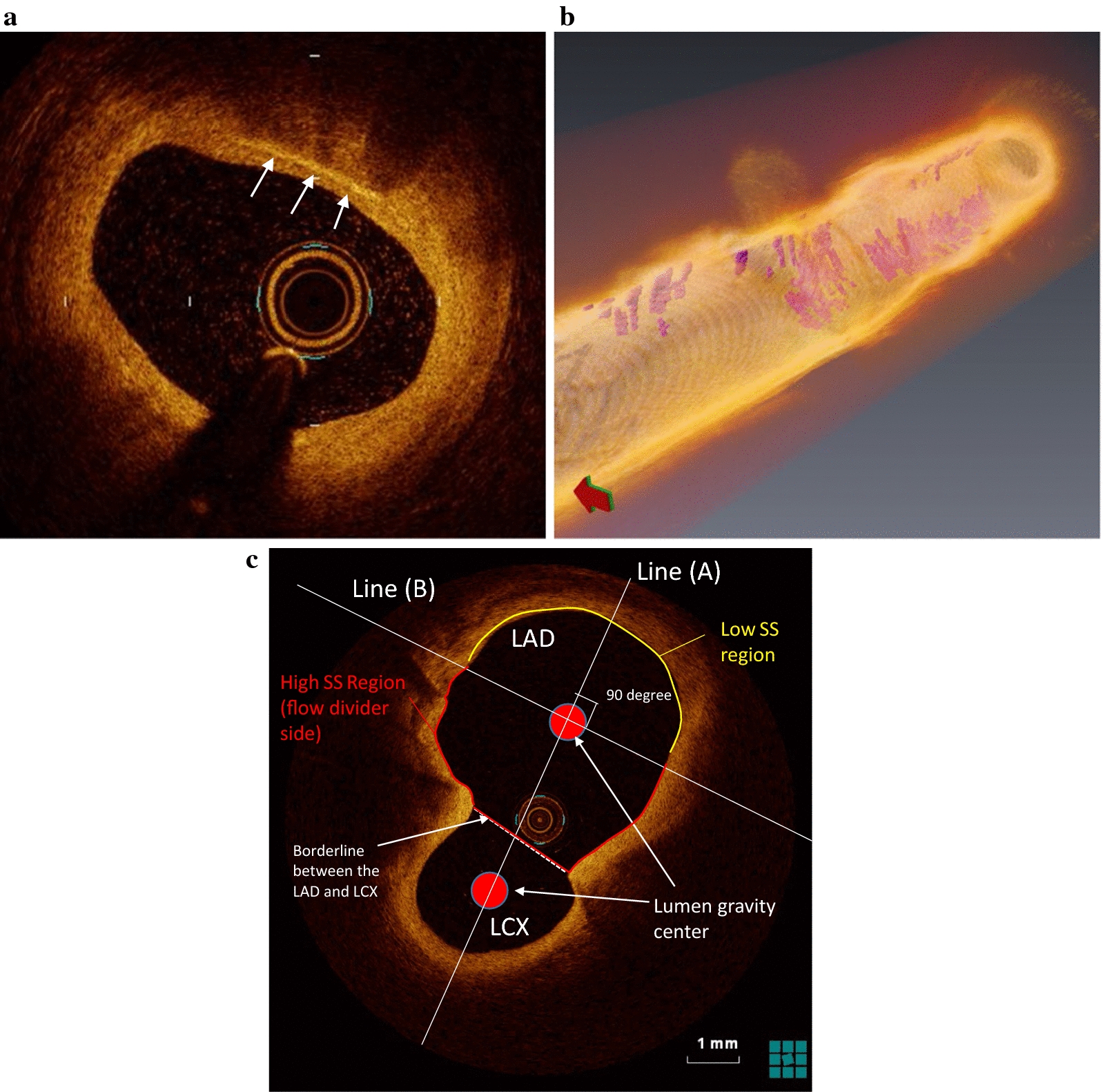
OCT analysis of macrophage accumulation. **a** Representative two-dimensional image. A line-like high luminance within coronary having a striped shadow is considered as macrophage accumulation (arrows). The cross-sectional areas of the high luminance were measured to calculate macrophage accumulation volume using consecutive frame by frame OCT images within the proximal LAD segment of interest. **b** Representative three-dimensional image. Macrophage accumulation is indicated by pink color. **c** Definition of high and low shear stress regions. See main text for detail. LAD, the left anterior descending artery; LCX, left circumflex artery; SS, shear stress

As measures on the tissue properties of the plaque segment examined by OCT, the minimum fibrous cap thickness as well as the maximum angular span of lipid core arc were determined [22]. In this study, the existence of macrophage accumulation was determined by OCT based on the presence of a line-like high luminance within the wall of the coronary artery having a striped shadow (Fig. 1a) [15]. In order to prevent contamination of artifacts, the findings were assumed to be recognized over three consecutive frames on the OCT. The identification of macrophage accumulation was automatically performed by extracting a region equal to or higher than a certain luminance with use of three-dimensional image analysis software AVIZO ™ (ver 6.2.1, MAXNET Corporation, Tokyo). In this analysis, erroneously extracted portions was excluded with visual inspection by an experienced observer. The area of macrophage accumulation was then displayed in 3D reconstructed image (Fig. 1b). Finally, the volume of the macrophage accumulation within the vascular segment of interest was automatically calculated from the summation of the area of each frame by the AVIZO software. The feasibility of the AVIZO in measuring 3D volume microstructure has been warranted in previous studies [23].

The coronary artery in the range of 20 mm from the LAD entrance part was next divided into two parts, the branch side half and the opposite side half, looking at the branching of the left circumflex (LCX) (Fig. 1c). At the LAD/LCX bifurcation cross-section, the centers of gravity of both blood vessel branches were first connected by a straight line (A). The straight line (B) orthogonal to the straight line (A) passing through the lumen gravity center of the LAD was then drawn. This line (B) divided the LAD wall into two regions on the LCX branch side and on the opposite side. The LCX side half, in other words, flow divider side, can be considered as a high shear-stress region, and the opposite side half as a low shear-stress region [24]. Considering the difference in observation range between the groups, normalized volume of the macrophage accumulation (per 1-mm-length) was also measured. Furthermore, macrophage accumulation density of total plaque and high- or low-shear stress region was also calculated by dividing the accumulated volume of the macrophage by each plaque volume of total and high- or low-shear stress region, respectively. The corresponded plaque volume was obtained from IVUS imaging, because visualization of the whole plaque is frequently difficult due to the limited penetration depth of the infra-red ray in OCT imaging.

### Statistical analysis

Values are shown as mean ± standard deviation, median [confidence interval (CI)], or number (percentages). For the comparison between the two groups, the χ^2^ test for the category variable and the t test for the continuous variable were performed. In the case where the continuous variable does not give a normal distribution, a Mann–Whitney test was used. In multivariate analysis, binary logistic regression analysis was performed where the explanatory variable which had a p < 0.2 in univariate analysis could be included. In this analysis, the presence of diabetes mellitus, hyper-uric-acidemia, value of HDL cholesterol, the minimum fibrous cap thickness, normalized total vessel volume and normalized plaque volume in the assumed low shear stress region and plaque volume were included. Statistical analysis was performed using IBM SPSS Statistics 24.0 (IBM, New York City, USA), and the p value less than 0.05 was determined to be statistically significant.

## Results

### Patients characteristics (Table 1)

Baseline characteristics per study group were summarized in Table 1. Average age and gender did not differ between the 2 groups. The frequency of ST elevation myocardial infarction was significantly higher in the non-DM group (p = 0.01), but those of non-ST elevation myocardial infarction and unstable angina were not significantly different between the two groups. The prevalence of coronary risk factors also did not differ between the two groups except for diabetes medicines. As for laboratory data, the value of HbA1c was significantly higher in the DM group than the non-DM group (8.0 ± 2.4% vs. 5.7 ± 0.2%, p = 0.001). However, no other laboratory items differed significantly between the two groups.

**Table 1:** Patients characteristics

	DM (n = 20)	non-DM (n = 20)	p value
Age (years)	64.2 ± 12.1	66.6 ± 15.9	0.593
Gender, male (%)	15 (75)	12 (60)	0.311
STEMI, n (%)	8 (40)	16 (80)	0.010
NSTEMI, n (%)	5 (25)	1 (5)	0.091
UAP, n (%)	7 (35)	3 (15)	0.137
Risk factor			
Hypertension, n (%)	13 (65)	10 (50)	0.337
Dyslipidemia, n (%)	10 (50)	12 (60)	0.525
Hyperuremia, n (%)	4 (20)	1 (5)	0.342
Obesity, n (%)	8 (40)	4 (20)	0.168
Smoker, n (%)	11 (55)	8 (40)	0.342
Family history, n (%)	4 (20)	2 (10)	0.661
Medication			
Aspirin, n (%)	4 (20)	1 (5)	0.342
Calcium channel blocker, n (%)	6 (30)	7 (35)	0.736
ACEI/ARB, n (%)	9 (45)	4 (20)	0.091
Beta blocker, n (%)	4 (20)	0 (0)	0.106
Diuretics, n (%)	1 (5)	1 (5)	1.000
Statin, n (%)	5 (25)	3 (15)	0.695
Antidiabetic drug, n (%)	11 (55)	0 (0)	< 0.0001
Sulfonylurea, n (%)	4 (20)	0 (0)	0.106
DPP-4 inhibitor, n (%)	8 (40)	0 (0)	0.003
Insulin, n (%)	1 (5)	0 (0)	< 0.0001
Laboratory findings			
Hemoglobin (g/dl)	13.3 ± 2.2	13.0 ± 2.4	0.751
Creatinine (mg/dl)	0.93 ± 0.45	0.75 ± 0.16	0.116
BUN (mg/dl)	19.0 ± 11.7	14.1 ± 4.4	0.091
eGFR (ml/min/1.73 m^2^)	68.7 ± 23.6	76.6 ± 23.2	0.296
Total-C (mg/dl)	206.0 ± 50.7	206.8 ± 42.0	0.957
HDL-C (mg/dl)	39.8 ± 8.1	45.1 ± 13.7	0.157
LDL-C (mg/dl)	131.1 ± 44.8	132.5 ± 33.3	0.916
TG (mg/dl)	173.6 ± 119.9	111.1 ± 70.7	0.058
HbA1c(NGSP) (%)	8.0 ± 2.4	5.7 ± 0.2	0.001
UA (mg/dl)	5.8 ± 1.8	5.5 ± 1.5	0.550
hsCRP (mg/dl)	0.21 ± 0.15	0.21 ± 0.37	1.000

Data were expressed as number (percentage) or mean value ± standard deviation

### IVUS findings (Table 2)

The observation length was statistically comparable between the two groups (median value: 13.1 mm, CI 9.0–19.3 mm in the DM group and 16.5 mm, CI 14–20 mm in the non-DM group). The analysis by Gray scale IVUS showed that there was no significant difference in terms of IVUS measurement except for lumen volume, although normalized lumen volume (per 1-mm-length) was not significantly different between the groups. When comparing plaque volume in the high shear stress region and that low shear region between the groups, no significant difference was found regarding the absolute value as well as normalized value (per 1-mm-length).

**Table 2:** IVUS findings

	DM (n = 20)	non DM (n = 20)	p value
Segment length	13.1 (9.0–19.3)	16.5 (14.0–20.0)	0.063
Lumen volume (mm^3^)	81.5 (55.5–121.5)	138.1 (123.8–195.8)	0.004
Vessel volume (mm^3^)	225.7 (137.4–285.5)	269.0 (216.8–388.4)	0.096
Plaque volume (mm^3^)	131.3 (80.4–160.4)	129.1 (95.9–164.7)	0.925
Plaque volume of high SS region(mm^3^)	58.7 (35.6–73.7)	58.8 (35.9–68.9)	1.000
Plaque volume of low SS region(mm^3^)	63.5 (51.9–89.9)	67.1 (51.1–92.2)	0.620
Normalized lumen volume/1 mm (mm^3^/mm)	7.3 (5.8–9.2)	8.4 (6.8–11.2)	0.174
Normalized vessel volume/1 mm (mm^3^/mm)	16.5 (14.3–22.2)	15.4 (13.6–20.4)	0.758
Total normalized plaque volume (mm^3^/mm)	9.5 (7.4–12.0)	7.8 (6.1–9.7)	0.068
Normalized plaque volume in high SS region (mm^3^/mm)	4.3 (3.4–5.6)	3.2 (2.7–4.8)	0.072
Normalized plaque volume in low SS region (mm^3^/mm)	5.8 (3.5–6.9)	4.5 (3.4–5.5)	0.081

Values are expressed as median value (confidence interval). SS, shear stress

### OCT findings (Table 3)

The minimum fibrous cap thickness was significantly thinner in the DM group than the non-DM group (81.5 ± 17.3 μm vs. 99.5 ± 29.8 μm, p = 0.025). In the high shear stress region, the minimum fibrous cap thickness was not significantly different between the two groups. However, in the low shear stress region, this thickness was significantly thinner in the DM group than the non-DM group (92 ± 20.9 μm vs. 125 ± 29.8 μm, p < 0.0001). The maximum lipid core angle was not different between the two groups as for the entire vascular region of interest as well as the high or low shear stress regions.

**Table 3:** OCT findings

	DM (n = 20)	Non DM (n = 20)	p value
Fibrous cap thickness (μm)	81.5 ± 17.3	99.5 ± 29.8	0.025
Minimum fibrous cap thickness in high SS Region (μm)	94 ± 23.5	104 ± 39.8	0.339
Minimum fibrous cap thickness in low SS region (μm)	92 ± 20.9	125 ± 29.8	< 0.0001
Maximum lipid arc (degree)	146.6 ± 35.2	127.4 ± 27.8	0.063
Maximum lipid arc in high SS region (degree)	105.8 ± 32.6	85.5 ± 36.2	0.070
Maximum lipid arc in low SS region (degree)	119.8 ± 39.0	110.5 ± 37.1	0.446

Values are expressed as median value (confidence interval). SS, shear stress

### Characteristics of macrophage accumulation (Table 4)

When comparing indices between the DM and the non-DM groups, normalized volume of macrophage accumulation (per 1-mm-length) in the total vessel segment of interest as well as in the low-shear stress region was significantly larger in the DM group (p = 0.015 and p = 0.002, respectively) compared to the non-DM group. However, this value was not significantly different between the two groups in the high shear stress region (p = 0.999). Regarding macrophage density (per plaque volume), this value of the DM group had a higher tendency in the total vessel segment of interest (p = 0.060), and was significantly higher in the low-shear stress region (p = 0.003) compared to the non-DM group.

**Table 4:** Characteristics of OCT-derived macrophage accumulation in DM and non-DM patients with ACS

	DM (n = 20)	non DM (n = 20)	p value
Total normalized M-Vol (mm^3^/mm)	9.5 × 10^−3^(4.2 × 10^−3^ − 12.5 × 10^−3^)	2.9 × 10^−3^(1.4 × 10^−3 ^ − 11.3 × 10^−3^)	0.015
Normalized M-Vol in high SS region (mm^3^/mm)	1.9 × 10^−3^(0.4 × 10^−3^ − 7.8 × 10^−3^)	2.1 × 10^−3^(0.5 × 10^−3^ − 8.0 × 10^−3^)	0.999
normalized M-Vol in low SS region (mm^3^/mm)	4.0 × 10^−3^(2.2 × 10^−3^ − 9.7 × 10^−3^)	0.8 × 10^−3^(0.2 × 10^−3^ − 2.1 × 10^−3^)	0.002
p value between high SS and low SS regions	0.314	0.096	
Total M-density	9.9 × 10^−4^(4.1 × 10^−4^ − 12.9 × 10^−4^)	4.5 × 10^−4^(2.1 × 10^−4^ − 11.9 × 10^−4^)	0.060
M-density in high SS region	5.6 × 10^−4^(1.1 × 10^−4^ − 18.8 × 10^−4^)	7.1 × 10^−4^(1.4 × 10^−4^ − 16.5 × 10^−4^)	0.779
M-density in low SS region	6.5 × 10^−4^(4.5 × 10^−4^ − 15.1 × 10^−4^)	1.5 × 10^−4^(0.5 × 10^−4^ − 4.5 × 10^−4^)	0.003
p value between high SS and low SS regions	0.565	0.030	

Values are expressed as median value (confidence interval). M-vol, macrophage accumulation volume; SS, shear stress; M-density, macrophage accumulation density per IVUS-derived plaque volume

**Fig. 2:**
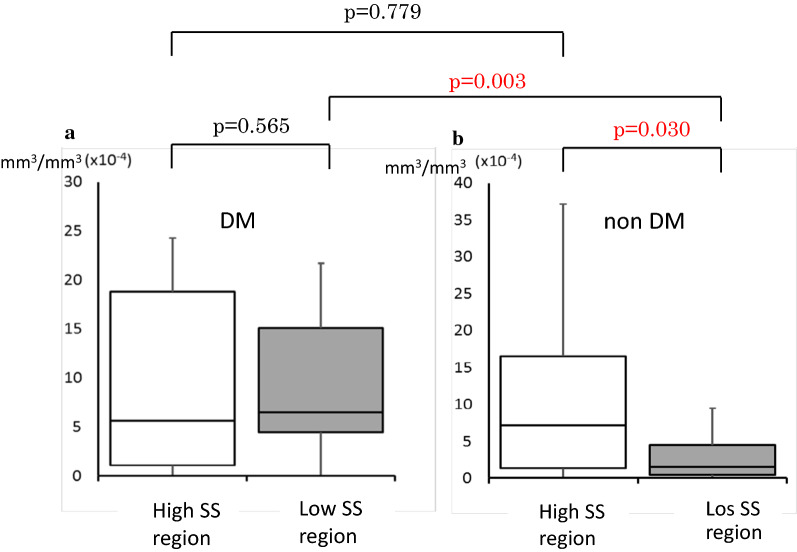
Comparisons of macrophage density per plaque volume between high and low shear stress regions, and between diabetic and non-diabetic patients. DM, type 2 diabetes mellitus; SS, shear stress

On the other hand, when comparing each index between the high and the low shear stress regions (Fig. 2), normalized volume of macrophage accumulation of the non-DM group had a larger tendency (p = 0.096), and its macrophage density was significantly higher (p = 0.030) in the high shear stress region. However, both indices of the DM group were comparable between the regions (p = 0.314 and p = 0.565. respectively).

On the basis of the data above, a multivariate analysis in order to reveal significant determinants for the distribution pattern of macrophage accumulation was performed. The objective value used [(macrophage accumulation density in high shear stress region) minus (macrophage accumulation density in low shear stress region)]/(accumulation density of macrophage in the whole region). This index was formed, because some plaque had no macrophage infiltration at all and simple calculation of the ratio of macrophage accumulation volume between the two sides was difficult in such cases. As a result, only the presence of diabetes mellitus was a significant regulatory factor (p = 0.033) (Table 5).

**Table 5:** Multivariate analysis for determinant of macrophage accumulation distribution pattern

Variables	Odds ratio	p value
Diabetes mellitus	0.086	0.033
Hyperuremia	0.278	0.338
HDL-C	0.993	0.853
Minimum fibrous cap thickness in low SS region	0.97	0.069
Normalized plaque volume in low SS region	0.086	0.777
Total normalized vessel volume	0.777	0.060

The objective value of macrophage accumulation distribution pattern is calculated from [(macrophage accumulation density in high shear stress region) minus (macrophage accumulation density in low shear stress region)]/(accumulation density of macrophage in the whole region). SS, shear stress

## Discussion

The major finding of the present study with using three-dimensional OCT and IVUS was that macrophage accumulation was more frequent in DM patients with ACS compared to non-DM patients, even indicating that its distribution pattern was significantly different in such patients. Macrophage was more accumulated in the flow-divider (higher shear stress) region in non-DM patients with ACS than the opposite (lower shear stress) region. However, the difference was significantly attenuated in DM patients with ACS, while macrophage infiltration was more frequent within the opposite (lower shear stress) region, suggesting homogeneous distribution of macrophage accumulation along the coronary wall. Furthermore, among various risk factors the presence of DM might play a pivotal role in the distribution pattern of macrophage accumulation, according to a multivariate analysis.

### Diabetes and macrophage accumulation

Difference in tissue characteristics of coronary plaque between DM and non-DM patients have been reported in many previous necropsy or in vivo angiography, IVUS and OCT studies [25–32]. Necropsy examinations have shown that DM patients had a greater necrotic core, a thinner fibrous cap, increased macrophage infiltration and healed ruptured plaque within coronary arterial wall compared to non-DM patients [25, 26, 33]. Among in vivo studies, coronary angiographic studies have shown that coronary artery lesion is more severe, extended, diffuse and multivessel in DM patients [27]. IVUS examinations have revealed that coronary plaque of DM patients is more abundant with a larger necrotic core and limited vascular remodeling [28–30, 34, 35]. More recently, studies with use of OCT have shown that DM patients had a larger lipid arc, a thinner fibrous cap, and a higher prevalence of calcification and mural thrombus [31, 32, 36, 37]. The results of our own support these data; the normalized plaque volume tended to be greater, and the maximum lipid arc tended to be greater in patients with DM compared with those without in our study. Furthermore, our data uniquely showed characteristics of macrophage accumulation pattern in DM patients with ACS, suggesting more abundant as well as more homogeneous macrophage infiltration within coronary wall, compared to non-DM patients. Previous studies have shown that DM patients have likely more abundant macrophage accumulation within coronary arterial wall [16–18], however, these studies did not examine difference in the distribution pattern of macrophage infiltration.

Increased inflammatory markers and mediators have been observed in patients with type 2 DM [38–40]. Increased and activated macrophages in DM patients express scavenger receptors to more aggressively engulf oxidized low-density lipoprotein followed by forming foam cells and leading to production of chemokine to promote atherosclerosis. Furthermore, cholesterol crystals captured by macrophage yields inflammasome complex activation [41] producing interleukin-1 beta to provide a feed forward mechanism to amplify atherosclerosis. In DM patients, all of these may be accelerated [25, 42, 43], which may support our data in which macrophage accumulation was more increased in DM patients.

### Shear stress and macrophage accumulation

The distribution of macrophage accumulation may be mainly determined by heterogeneity of shear stress on the coronary intimal surface. It has been well known that atherosclerotic plaque volume progression can be observed more frequently in a lower shear stress region [44, 45]. However, the role of shear stress in plaque destabilization is under controversy, but considerable number of reports have documented that high shear stress plays an important role in plaque destabilization [46–50], including enhanced extracellular matrix degradation [51, 52], thinning of fibrous cap [51–53] and increasing of necrotic core [53]. The present study also provided the similar observations, for example, plaque volume appeared to be greater in low-shear stress regions compared with high-shear stress regions, while lipid arc appeared to be greater in high-shear stress regions compared with low-shear stress regions, although it did not reach statistical significance. Moreover, macrophage has been found to be more frequently accumulated in a higher shear stress region in vascular wall [10–14], which is comparable to the data of our own in non-DM patients.

Some discrepancies between low shear stress theory that enhances plaque progression and high shear stress that enhances macrophage accumulation and plaque destabilization may be explained as follows: High shear stress actually enhances endothelial NO secretion which has been shown to have anti-inflammatory effects [54], however, it has been shown that high shear stress induces increased expression of endothelial adhesion molecules, such as ICAM and VCAM, resulting in enhanced monocyte adherence followed by increasing macrophage formation within atherosclerotic plaque [10, 53]. Therefore, plaque tissue modification may be determined by the balance among these contradictory factors.

In the present study, macrophage was accumulated within coronary wall more homogeneously regardless of shear stress degree in DM patients with ACS. It was especially interesting that macrophage accumulation was more enhanced in the lower shear stress region in DM patients with ACS. This may be not only due to a change of the balance described above, but also due to substantial endothelial dysfunction, which has been commonly observed in DM patients [42, 43, 55]. Significant endothelia dysfunction in DM may deteriorate shear-stress-dependent mechanism of determining coronary tissue characteristics followed by homogeneous macrophage accumulation.

### Study limitations

There are limitations in the present study. This study was a single-center, retrospective study. The number of patients examined was relatively small, and assessment of macrophage accumulation was limited to LAD segment where the influence of curvature was considered to be relatively small. Clinical profiles except diabetic parameters between DM and non-DM group could not be completely matched, for example, STEMI was more frequent in non-DM patients than in DM ones, and this limitation should be considered in interpreting our data. In addition, the target vessel segment was divided into two parts, flow divider side and the opposite side, which were assumed to be a higher and a lower shear stress regions, respectively [24]. However, the absolute value of shear stress was not measured, the measurement of which was actually hardly possible in clinical settings.

In this study we detected macrophage accumulation within coronary arterial wall not by direct histopathological way but by in vivo OCT which had some inherent limitations [22], which should be considered in interpreting out results.

## Conclusions

Macrophage accumulation was more abundant and homogeneous within coronary arterial wall in DM patients with ACS compared to non-DM patients, suggesting that plaque destabilization may occur more widely throughout coronary wall in DM patients.

## Data Availability

The datasets used and/or analysed during the current study are available from the corresponding author on reasonable request.

## Abbreviations

ACS: Acute coronary syndrome
CAG: Coronary angiography
DM: Diabetes mellitus
IVUS: Intravascular ultrasound
LAD: Left anterior descending branch
LCX: Left circumflex branch
LDL: Low-density lipoprotein
OCT: Optical coherence tomography
PCI: Percutaneous coronary intervention
3D: 3-dimensional
